# Enhanced dye removal using montmorillonite modified with graphene quantum dots in sustainable salep nanocomposite hydrogel

**DOI:** 10.1038/s41598-024-57729-0

**Published:** 2024-03-25

**Authors:** Kolsum Mohammad Sharifi, Ahmad Poursattar Marjani, Peyman Gozali Balkanloo

**Affiliations:** Department of Organic Chemistry, Faculty of Chemistry, Urmia University, Urmia, Iran

**Keywords:** Biopolymer, Quantum dot graphene, Montmorillonite, Hydrogel, Dye removal, Semi-IPN, Biopolymers, Pollution remediation

## Abstract

This research investigated the utilization of graphene quantum dot/montmorillonite (GQD/MMT) as an effective nanofiller in a hydrogel composed of salep biopolymer. The semi-IPN hydrogel was synthesized using salep as the substrate, acrylamide (AAm) as the monomer, ammonium persulfate (APS) as an initiator in free radical polymerization, and *N*,*N*'-methylenebisacrylamide (MBA) as a cross-linking agent. The hydrogels were applied to remove safranin (SA), methylene blue (MB), crystal violet (CV), methyl green (MG), congo red (CR), and malachite green (MG) dyes from the water. The diverse properties were analyzed using a scanning electron microscope, fourier infrared spectroscopy, mapping, energy dispersive spectroscopy, weighing analysis, X-ray diffraction, and thermal stability analyses. The optimism of the prepared adsorbent in dye absorption was evaluated by measuring the swelling amount, pH impact, adsorbent dosage, and contact time. The adsorption calculations were described using kinetics and isotherm models. The results indicated that the Langmuir isotherm model (R^2^ = 99.6) and the pseudo-second-order kinetic model (R^2^ = 99.9) provided the best fit for the absorption process of MB. The presence of additional amounts of GQD/MMT had a reciprocal effect on the adsorption efficiency due to the accumulation of GQD/MMT in the semi-interpenetrating polymer network (semi-IPN (structure. The findings revealed that the samples exhibited high thermal stability, and the absorption process was primarily chemical. Furthermore, the nanocomposite hydrogels demonstrated distinct mechanisms for absorbing anionic dye (CR) and cationic dye (MB). Under optimal conditions, using 7 wt% GQD/MMT at a concentration of 5 ppm, pH = 7, an adsorbent dosage of 50 mg, at room temperature, and a contact time of 90 min, the maximum removal efficiencies were achieved: MB (96.2%), SA (98.2%), MG (86%), CV (99.8%), MG (95.8%), and CR (63.4%). These results highlight the adsorbent's high absorption capacity, rapid removal rate, and reusability, demonstrating its potential as an eco-friendly and cost-effective solution for removing dyes from water.

## Introduction

Due to the rising global population, growing water demand, and unsustainable water resource management, the global freshwater requirement has escalated. Half the global population lacks access to safe drinking water sources. If the current trend persists until 2050, when the world's population is projected to reach 10 billion people, this number could triple^1^. Households and various industries, such as mining, paint, plastic, textile, paper, food, fertilizer, pesticides, cosmetics, pharmaceuticals, electronics, plating, wood, and leather, produce large volumes of wastewater^2,3^. Among the types of pollution, the pollution of water sources with toxic dyes has caused great concern. Which are widely used in various industries, and a large amount of colored wastewater is produced^4,5^. Dyes are not biodegradable, so these pollutants cannot be removed naturally from the environment^6–8^.

Researchers have extensively studied various biological, chemical, and physical approaches to water purification. These methods include chemical precipitation, coagulation^9^, photocatalysis^10–12^, membrane filtration^13^, osmosis^14^, surface absorption^4,15–17^, biological processes^18^, oxidation^18^, and more. Among these techniques, surface absorption is widely acknowledged as a viable method for treating wastewater containing dyes. This method is favored due to its cost-effectiveness, non-toxicity resulting from product formation, ease of operation, non-hazardous nature, and scalability for large-scale applications^19–21^. Various adsorbents such as clay, carbon nanotubes, metal–organic frameworks, carbon nanofibers, metal oxides, and polymers have been developed and used in wastewater treatment^22–24^. Natural polymers and their derivatives are extensively utilized in various industries. Their widespread use is attributed to their natural abundance and renewable nature, making them highly desirable. These natural polymers can be instrumental in developing advanced materials, taking the form of films, membranes, coatings, hydrogels, and micro and nanoparticle systems^25^.

Hydrogels are polymer networks with hydrophilic properties, a three-dimensional structure, and a high ability to absorb water^26^. Hydrogels have a porous structure and functional groups, making them desirable adsorbents for removing water contaminants^27^. Adsorbents made from hydrogels exhibit high efficiency in removing dyes. The water-attracting pores within the hydrogels enable the smooth diffusion of water molecules across the interconnected structure. This creates a conducive environment for dissolved dyes to interact with the active functional groups along separate polymer chains^28–31^. Hydrophilic functional groups present in the hydrogels network, such as SO_3_H, CONH_2_, CONH, NH_2_, OH, and COOH groups, along with the capillary forces within the pores, contribute to the hydrophilicity of hydrogels. This hydrophilicity enables the hydrogel to undergo swelling when exposed to aqueous solutions^32^. Hydrogels provide a wide range of flexibility regarding synthesis methods, rapid swelling behavior, and adjustable surface properties. They can be tailored to possess specific characteristics such as charge, functionality, and the ability to facilitate fast diffusion processes. Hydrogels also offer a large surface area, controllable pore structure, catalytic properties, permeability, and thermal stability. These attributes make hydrogels versatile materials with diverse applications in various fields^33,34^.

Recently, efforts have been made to improve the absorbent properties of hydrogels, such as mechanical and chemical stability, absorption rate, and capacity. Semi-IPN hydrogels were synthesized by blending a cross-linked polymer with another polymer to create a new network. The resulting hydrogels exhibit excellent absorption capacity, high resistance to water and salt, and superior mechanical strength. These characteristics make semi-IPN hydrogels a promising candidate for removing dyes from wastewater. The semi-IPN hydrogels were tested for their ability to remove dyes from water, and the results showed that they effectively removed various dyes^35^. The high removal efficiency of semi-IPN hydrogels is attributed to their unique structure, which provides a large surface area for adsorption and a porous structure for the diffusion of dyes^36^. The swelling capacity of hydrogels indicates their absorption capacity, which can be influenced by factors such as intermolecular spaces in the three-dimensional network, the presence of hydrophilic groups on the polymer backbone of the hydrogel, and the size of pores on the hydrogel surface. Therefore, parameters such as the amount of initiator, polymer, monomers, and solvent volume are optimized during hydrogel preparation to achieve optimal conditions for an ideal hydrogel^37,38^.

Nanocomposite hydrogels are highly hydrated polymer networks, also called hybrid hydrogels. These hydrogels can be engineered to possess exceptional physical, chemical, electrical, and biological properties^39^. In addition to their advantages, hydrogels have notable drawbacks, primarily their low mechanical strength and elastic properties^40^. Polymer matrices reinforced with nanofillers have garnered significant attention from researchers as a means to enhance their mechanical strength. Nanofillers such as clay, graphene, MMT, and carbon nanotubes are utilized to reinforce the polymer matrix, resulting in hydrogel nanocomposites^41^.

GQDs, a recently discovered carbon nanomaterial, are carbon-based materials derived from two-dimensional graphene. This two-dimensional structure restricts electronic transport in all three spatial dimensions. GQDs possess small dimensions, with a diameter of less than 20 nm (< 20), allowing them to trap excitons effectively^42^.

GQDs exhibit a crystal lattice structure resembling a bee tulip, with carbon atoms forming hexagonal rings. This arrangement gives rise to the sp^2^ hybridized characteristics of GQDs, which facilitate the transfer of electrons in π orbitals^43^. GQDs possess essential properties, including low toxicity, stable fluorescence, and surface bonding with inert chemicals. They also have a high quantum yield, making them suitable for bio-imaging, light-emitting diodes, photoelectrocatalysis, drug delivery, sensors, and pollutant absorption^44^. The poor water penetration of GQDs in an aqueous solution presents challenges in their separation. To address this, researchers have focused on immobilizing GQD nanoparticles onto the surface of materials such as gold and double-layer hydroxides. Additionally, the hydrogen bond interaction between the hydroxyl group of GQDs and dye molecules is relatively weak, making it difficult to adjust the surface charges for a stronger electrostatic attraction and effective removal of cationic dyes. Therefore, to enhance the adsorption rate of cationic dyes, GQDs are immobilized on a base material that can rapidly dissociate and adjust the surface charge^45^.

MMTs possess several interesting properties, including strong hydrophilicity, excellent cation exchange capacity, and a large surface area^46^. Its dihedral structure characterizes MMT. The crystal structure of MMT consists of stacked layers with a thickness of approximately 1 nm^47^. MMT possesses a net negative charge in its layered structure, which arises from the isomorphic replacement of aluminum with magnesium in the octahedral sheets and silicon with aluminum in the tetrahedral sheets. This substitution leads to a negative charge within the MMT structure^48^. Indeed, due to its net negative charge and large surface area, MMT is widely recognized for its high adsorption capacity for cationic contaminants. The negatively charged surface of MMT attracts and adsorbs cations, making it an effective adsorbent for various cationic pollutants^49,50^.

Significant research has been conducted in developing absorbents and exploring new and effective methods for removing various dyes. Mu et al. utilized a nanocomposite called Mt/PANI/Fe_3_O_4_^2^ to adsorb the cationic dye MB^51^. Amari et al. investigated the synthesized Cs/N-GQD nanocomposite for its efficacy in removing various pollutants, including dyes, bacteria, and others^52^. Peighambardoust et al. employed CMC-g-P(AAm/MMT), prepared through the free radical method, to remove MlG dye from an aqueous solution^53^. Mahmoud et al. investigated ZnO/C-Foam/GQDs/Alginate dots that were synthesized to remove MB cationic dye^54^.

This study synthesized a semi-IPN network to create a nanocomposite hydrogel structure. The nanofillers used in the synthesis included acrylamide, salep, and GQD/MMT, with varying weight percentages. The free radical polymerization method was employed to prepare the nanocomposite hydrogel.

## Experimental

### Materials

Salep powder has been obtained locally. MMT and AAm were purchased from Millipore Sigma, USA. MBA, APS, ethanol, NaOH, and citric acid monohydrate (CA) were provided by Merck. All dyes used were purchased from Merck. Deionized water was used as a solvent or to prepare a solution.

### Synthesis of GQD

**Figure 1:**
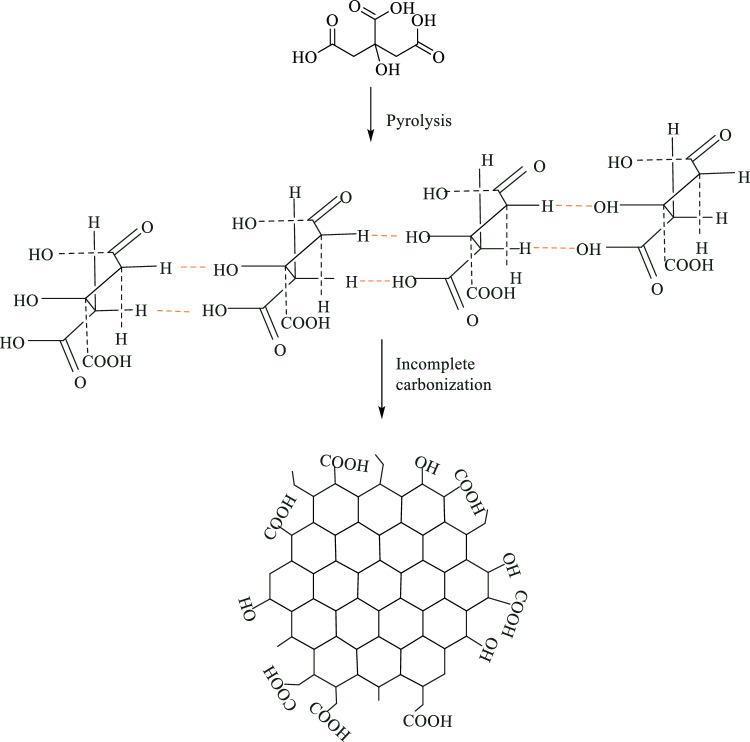
A diagram of GQDs formation from CA.

The preparation of GQDs was done through the direct pyrolysis of CA (Fig. 1). To prepare GQDs, 2 g of CA was added to a beaker at 200 °C to convert the white solid CA into an orange liquid. Then, 20 mL of NaOH solution (0.25 M) was added dropwise under vigorous stirring. The synthesized GQD solutions can be stored at 4 °C for 30 days^55,56^.

### Preparation of GQD/MMT

3 g of MMT was dispersed with distilled water (60 mL) using a magnet for 30 min. Subsequently, the orange liquid containing 12 mL of GQD was added stepwise to the 60 mL MMT suspension prepared at 80 °C. The resulting mixture was allowed to react for 1 h in an oil bath and was kept at room temperature for 2 h. The pH of the obtained sediment was neutralized, and afterwards, it was dried at 100 °C in a drying oven^45^.

### Synthesis of semi-IPN hydrogel nanocomposite

A salep-based nanocomposite hydrogel was prepared using a dispersion of GQD/MMT nanohybrid. 500 mg of salep powder and 15 mL of water were placed in a lab balloon and dissolved in an 80 °C oil bath using a magnetic stirrer. Subsequently, 0, 1, 3, 5, and 7 wt % of GQD/MMT (coded as samples in Table 1) were added to the solution. After 20 min, 2 g of AAm dissolved in 5 mL of water was added. Then, MBA (100 mg)and APS (30 mg) initiators were added with 5 mL of water and dissolved through vigorous stirring. Finally, the obtained hydrogel was washed with ethanol for 24 h, followed by oven-drying for 24 h at a temperature of 85–90, and pulverized into powder form.

**Table 1:** Amount of adsorbents used in each sample.

Sample code	GQD/MMT weight percentage (%)
GQD/MMT_0_	0
GQD/MMT_1_	1
GQD/MMT_3_	3
GQD/MMT_5_	5
GQD/MMT_7_	7

## Characterizations

The adsorbent's characteristics were investigated using various techniques: Fourier Transform Infrared Spectroscopy (FTIR) (Bruker IFS-66/S FTIR) in the range of 400–4000 cm^−1^ to identify functional groups; Field Emission Scanning Electron Microscope (FESEM) (Hitachi (S4160) FE-SEM) for imaging and determining surface characteristics and morphology; Energy-Dispersive X-ray Spectroscopy (EDS) analysis performed by TSCAN Electron Microscope with a copper target for elemental analysis; XRD analysis was carried out using X’Pert PRO MPD PANalytical Compan instrument; Thermal Gravimetric Analysis (TGA) (LINSEIS, STA PT-1000, Germany) to assess the samples' thermal stability between 298 and 1073 °K. The dye concentration in the treated solution was also measured using a UV–Vis spectrophotometer (CARRY100 Bio 5, China).

## Performance evaluation

### Measuring the swelling of absorbents

The swelling ability of hydrogels was measured at 25 °C. A specific amount of dried hydrogels was placed in separate containers. Then, it was immersed in water to reach equilibrium. After one hour, the excess water was removed with filter paper to ensure that no excess water remained and weighed again; this process was repeated up to 6 times. Swelling (%) was measured using Eq. (1):

1 $${P}_{{\text{s}}}=\frac{{{\text{W}}}_{{\text{s}}}-{{\text{W}}}_{{\text{d}}}}{{{\text{W}}}_{{\text{d}}}}\times 100$$

The ${{\text{P}}}_{{\text{s}}}$ denotes swelling percentage, *W*_*d*_ and *W*_*s*_ refer to the dry adsorbent’s and swollen gel weights, respectively.

### Dye removal test

A dye solution containing six dyes (MB, MlG, CR, MG, CV, and SA) was prepared by dissolving each dye at a concentration of 5 ppm in deionized water. Next, 50 mg of the absorbent material was mixed with 25 mL of the dye solution, ensuring a constant dye concentration, and stirred for 90 min. Samples were collected from the mixture at regular intervals and centrifuged at 2000 revolutions for 10 min. The maximum absorption wavelength (λ_max_) for each dye and the visible absorption values at λ_max_ were measured using a dual UV–Vis spectrophotometer. Finally, the percentage of dye removal can be determined using the Eq. (2):

2 $$\mathrm{R }(\mathrm{\%})=\left(\frac{{{\text{C}}}_{0}-{{\text{C}}}_{{\text{t}}}}{{{\text{C}}}_{0}}\right)\times 100$$

*C*_*0*_ (mg/L) and *C*_*t*_ (mg/L) are the dye's initial and concentration times, respectively.

### Investigating practical factors in the absorption process

#### Effect of contact time on absorption

Equilibrium time is a crucial parameter in the design of an efficient wastewater treatment system. Longer contact times lead to increased absorption. The concentrations of 5 ppm of dyes were prepared to examine the impact of time on absorption. Additionally, 50 mg of the absorbent was mixed with 25 mL of the colored solution using a magnetic stirrer. The absorption process was conducted for 2 h, with measurements taken at 10-min intervals. The samples were analyzed using a spectrophotometer to investigate the changes in absorption over time.

#### Influence of additive dosage on absorption rate

In investigating dye removal employing solid adsorbents, the optimal quantity of additives plays a pivotal role. In order to fine-tune the weight of the absorbent within hydrogels with different proportions of GQD/MMT, 50 mg samples were meticulously prepared in 25 mL of a solution containing dye, utilizing a magnetic stirring apparatus. This process aimed to assess the impact of varying additive dosages on the absorption rate.

### Properties of semi-IPN hydrogel nanocomposite

The morphology of the hydrogel nanocomposite was analyzed using a FESEM scanning electron microscope (ZEISS Sigma 300) along with EDS and mapping. The chemical structure of the additives was determined through fourier transform spectroscopy (Bruker IFS-66/S FTIR) in the wavelength range of 400–4000 cm^−1^. XRD patterns were obtained using a Philips PW 1730 instrument with Cu k_α radiation (1.5405 Å) to assess the crystallite size and phase purity. The thermal stability of the prepared nanocomposite hydrogels, specifically the semi-IPN and their properties, was investigated using TGA and DSC techniques. These analyses were performed using a LINSEIS STA PT-1000 instrument in a nitrogen environment. The temperature range for TGA was 40–800 °C, while DSC measurements were conducted over the same temperature range.

## Results and discussion

### Synthesis mechanism of semi-IPN hydrogel nanocomposite

**Figure 2:**
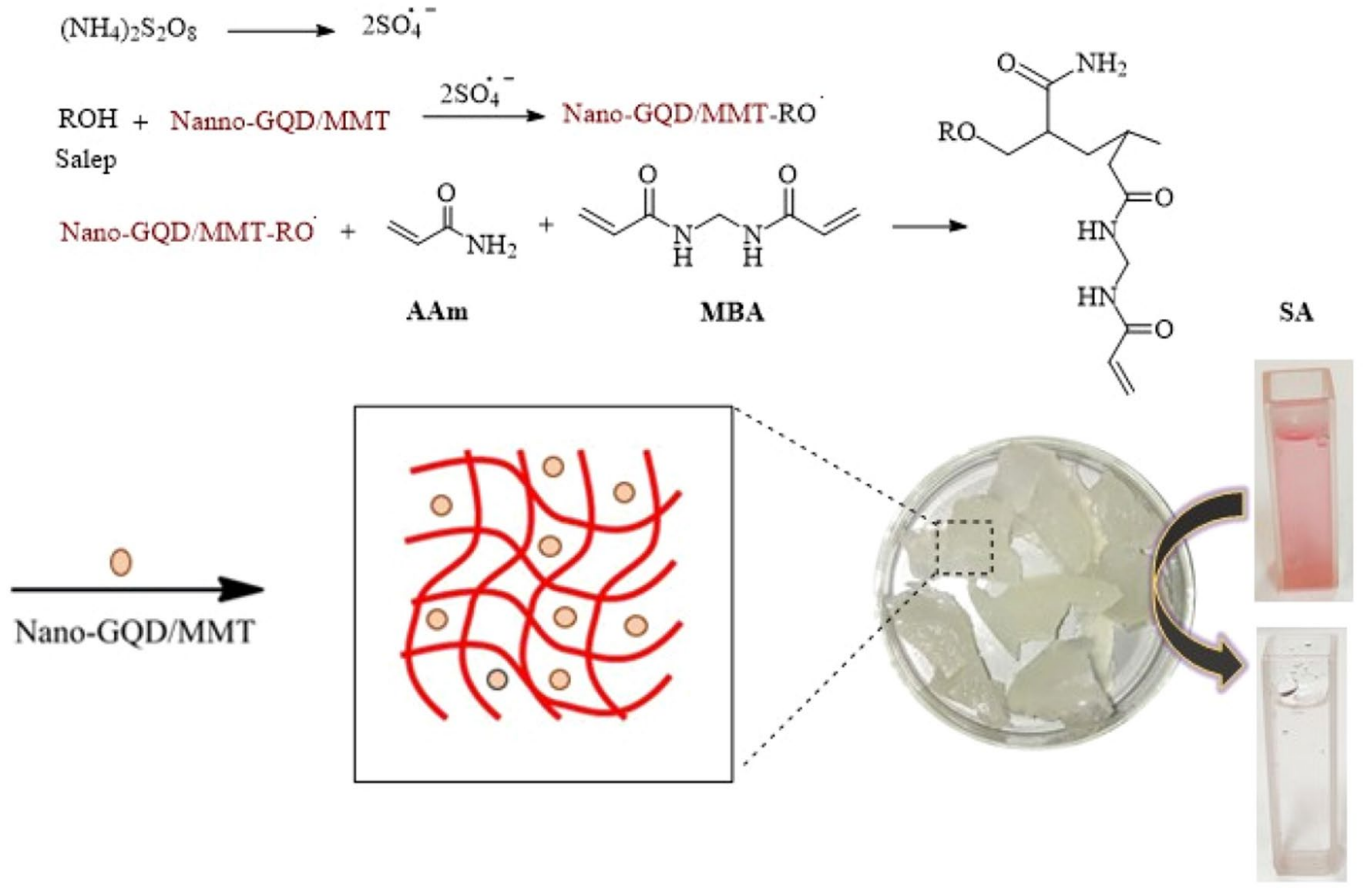
The mechanism of acrylamide hydrogel synthesis is based on salep.

In Fig. 2, the cross-linking and polymerization of acrylamide bonds occur on the salep backbone in an aqueous environment. This process utilizes APS as a free radical initiator and MBA as a cross-linking agent. When heated to 75 °C, persulfate undergoes decomposition, generating sulfate anion radicals. These radicals’ abstract hydrogen atoms from the hydrogen and hydroxyl groups in the salep backbone. This leads to breaking C–H or O–H bonds in the polysaccharide chain. In the presence of the MBA networking agent, cross-linking occurs, forming transverse connections. The salep filaments enter the polymer matrix and contribute to developing a semi-IPN hydrogel lattice through additional hydrogen interactions. To fabricate the network structure of the final semi-IPN hydrogel nanocomposite, the combined GQD/MMT is employed as a physical cross-linker^32,57^.

### FTIR analysis

**Figure 3:**
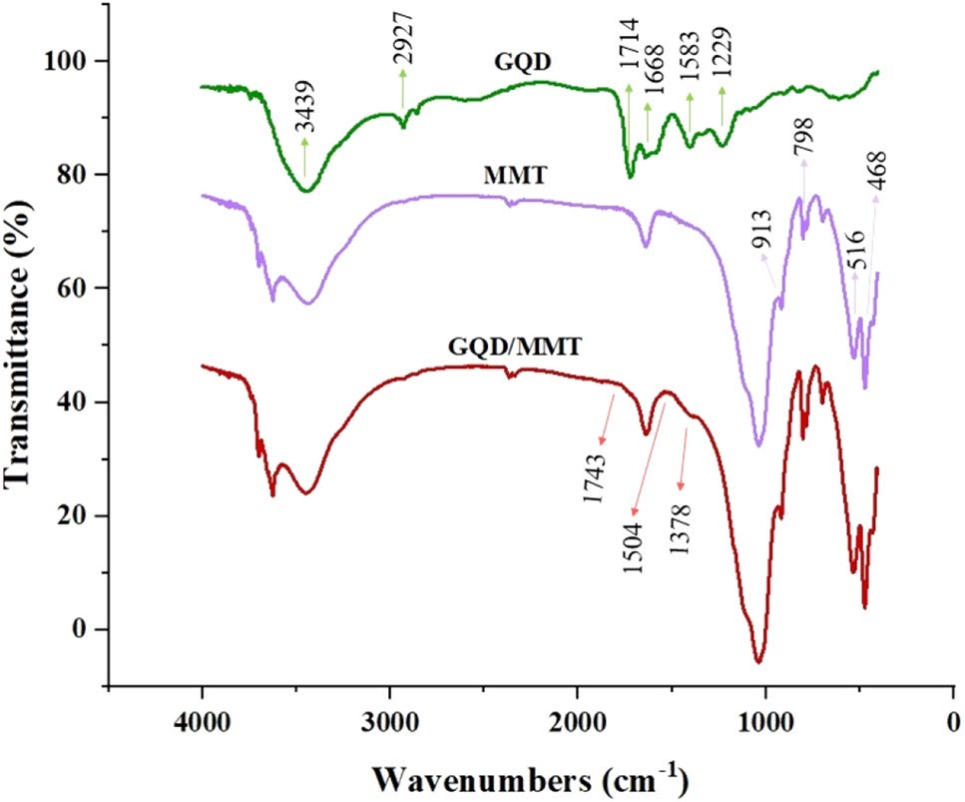
FTIR spectra of GQD, MMT, and GQD/MMT.

In the MMT spectrum, specific bands were assigned as follows: the band at 913 cm^−1^ relates to the vibrations of Al–Mg–OH, the 798 cm^−1^ relates to the vibrations of Al–Al–OH, the 526 cm^−1^ corresponds to the vibrations of Si–O–Fe, and the 468 cm^−1^ related to the vibrations of Si–O–Al^58,59^. The broad peaks at 3439 cm^−1^ in the GQD spectrum are assigned to –OH stretching bands. Furthermore, the peak at 2927 cm^−1^ in the spectrum indicates asymmetric stretching and symmetrical C–H vibrations. The 1714 cm^−1^ is related to the bending vibrations of the C=C group. The 1668, 1583, and 1229 cm^−1^ were assigned to the functional groups C=O, C–O (carboxy), and C–O (alkoxy), respectively^60^. The low intensity of these peaks in the GQD spectrum confirmed the reduction of ketone, hydroxyl, and epoxide groups during the formation of GQD^61^. The changes in functional groups of GQD/MMT were investigated. It can be observed that the water absorption leads to the presence of –OH vibration peaks at 3442 and 1636 cm^−1^^62^. The indicative peaks of the carboxyl group's C=O vibration and the stretching vibration in the C=C plane of the aromatic ring are observed at approximately 1841 and 1504 cm^−1^, respectively. Furthermore, in the case of GQD/MMT samples, the peak observed at 1378 cm^-1^ can be attributed to the bending vibration of the C–H bond, providing evidence for the formation of GQD/MMT. (Fig. 3).

### XRD analysis

**Figure 4:**
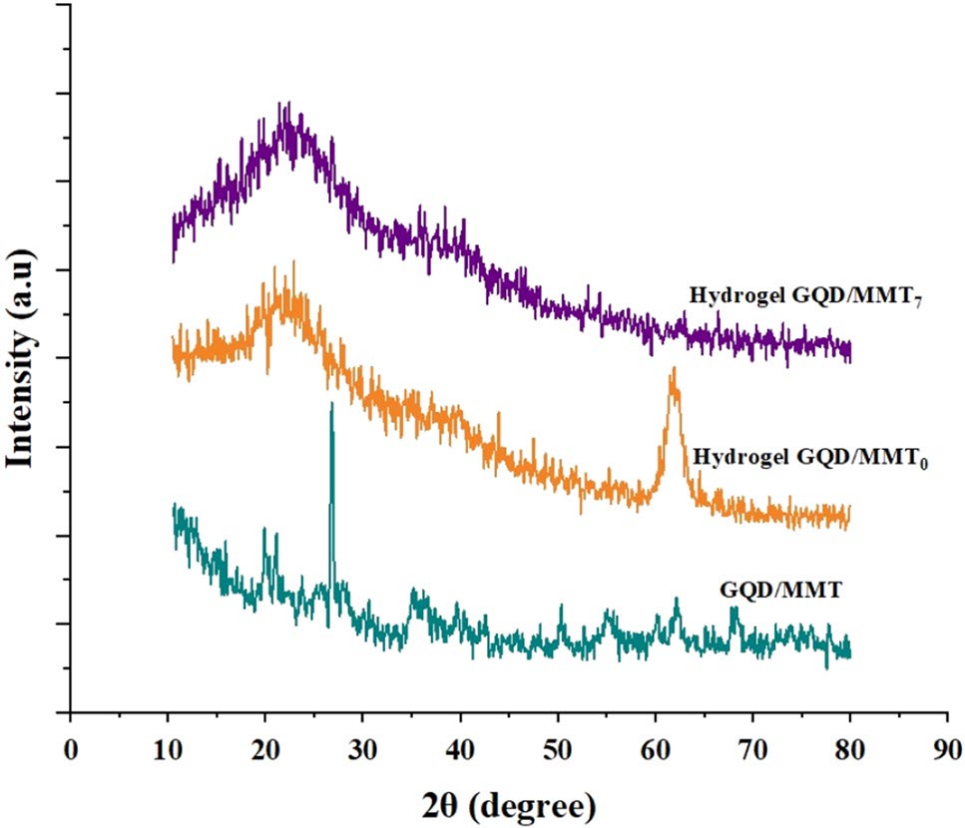
XRD patterns of the additive, neat, and modified hydrogel.

Figure 4 presents the XRD spectrum of GQD/MMT, hydrogel GQD/MMT_0_, and hydrogel GQD/MMT_7_. In the XRD spectrum of GQD/MMT, peaks observed at 21.25°, 26.8°, 35.33°, and 50.53° confirm the crystal structure of MMT. Additionally, based on the XRD analysis, the presence of quartz in the MMT structure is also observed, which has been previously reported by other researchers^63^. The absence of detectable signals for GQDs in the XRD spectrum can be attributed to their low quantities, high dispersal, and low crystallinity within the GQD/MMT composite. This suggests that the parent crystal structure of MMT remains intact even after the deposition of GQDs on its surface. In the XRD spectrum of hydrogel GQD/MMT_0_, the highest intensity peak appears at 22.45° (2θ scale), indicating partial crystallinity of the hydrogel with a predominant amorphous phase. Another peak at 62.10° is also observed. In the synthesized hydrogel GQD/MMT_7_ spectrum, the intensity of the peaks decreases, indicating a reduction in the crystallinity of the hydrogel upon the addition of GQD/MMT. This reduction can be attributed to the formation of new interactions between GQD/MMT and the hydrogel, which may disrupt the interactions among hydrogel chains. Furthermore, in the hybrid hydrogel, the peak at 2θ = 22.2° is broadened, suggesting an enrichment of the amorphous structure in the hydrogel.

### TGA analysis

**Figure 5:**
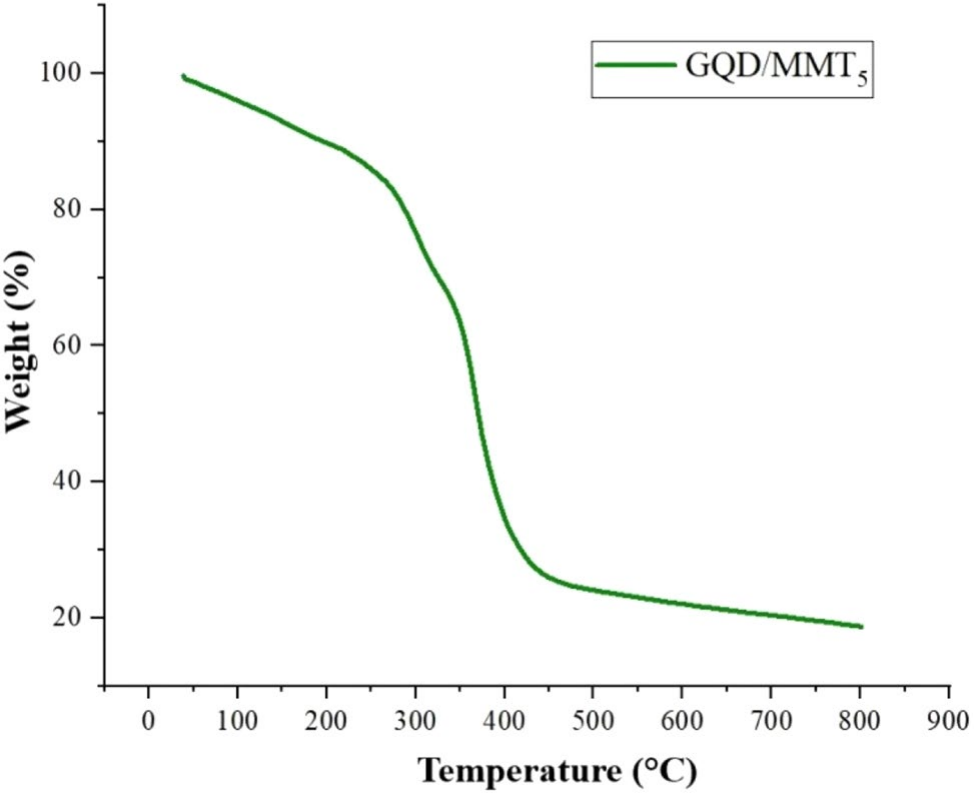
TGA thermograms of hydrogel nanocomposite semi-IPN containing 5 wt% of GQD/MMT.

Figure 5 shows the TGA curve of hydrogel GQD/MMT_5_ under nitrogen at above 800 °C. The thermogram shows similar weight loss trends in three stages. Initial decomposition is observed at 25–200 °C, which the evaporated polymer water may cause. The second reduction stage occurs at 280–200 °C due to the decomposition of amide and carboxylate groups in the polyacrylamide chain. The subsequent weight loss from 300 to 420 °C occurs due to the corruption of the semi-IPN spine. Finally, 20% of the mass of ashes will remain.

### FESEM and EDS analysis

**Figure 6:**
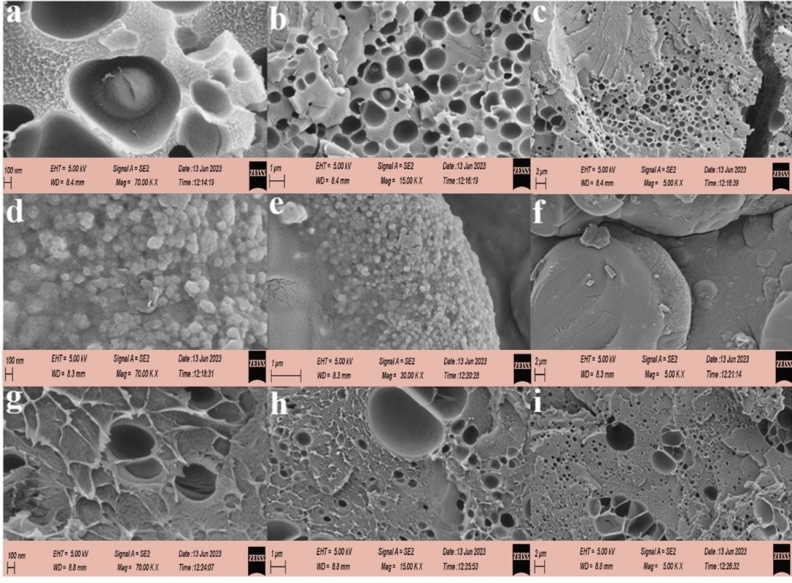
FESEM images of hydrogels GQD/MMT_0_ (**a**–**c**), GQD/MMT_3_ (**d**–**f**), and GQD/MMT_7_ (**g**–**i**).

FESEM (Field-Emission Scanning Electron Microscopy) analysis was conducted to investigate the surface characteristics and morphology of the prepared absorbents. Figure 6 displays the images obtained from the hydrogel without any additives (a–c), GQD/MMT_3_ hydrogel (d–f), and GQD/MMT_7_ hydrogel (g–i). The results indicate that adding 3 wt% of GQD/MMT to the absorbent structure has resulted in a rough surface, which can be attributed to GQD/MMT within the absorbent. Moreover, the surface becomes even rougher as the GQD/MMT content increases to 7 wt%. The hydrogel GQD/MMT_7_ sample (g–i) exhibits the presence of wrinkles on its surface.

Furthermore, the absorbers' structure displays the formation of holes or voids, which are proportional to the content of GQD/MMT in the absorbent structure. These pores contribute to increased water diffusion and enhanced contaminant removal, making them advantageous for water absorption. Additionally, such wrinkled and rough pores can lead to a higher surface area of the adsorbent, potentially improving the adsorption efficiency.

**Figure 7:**
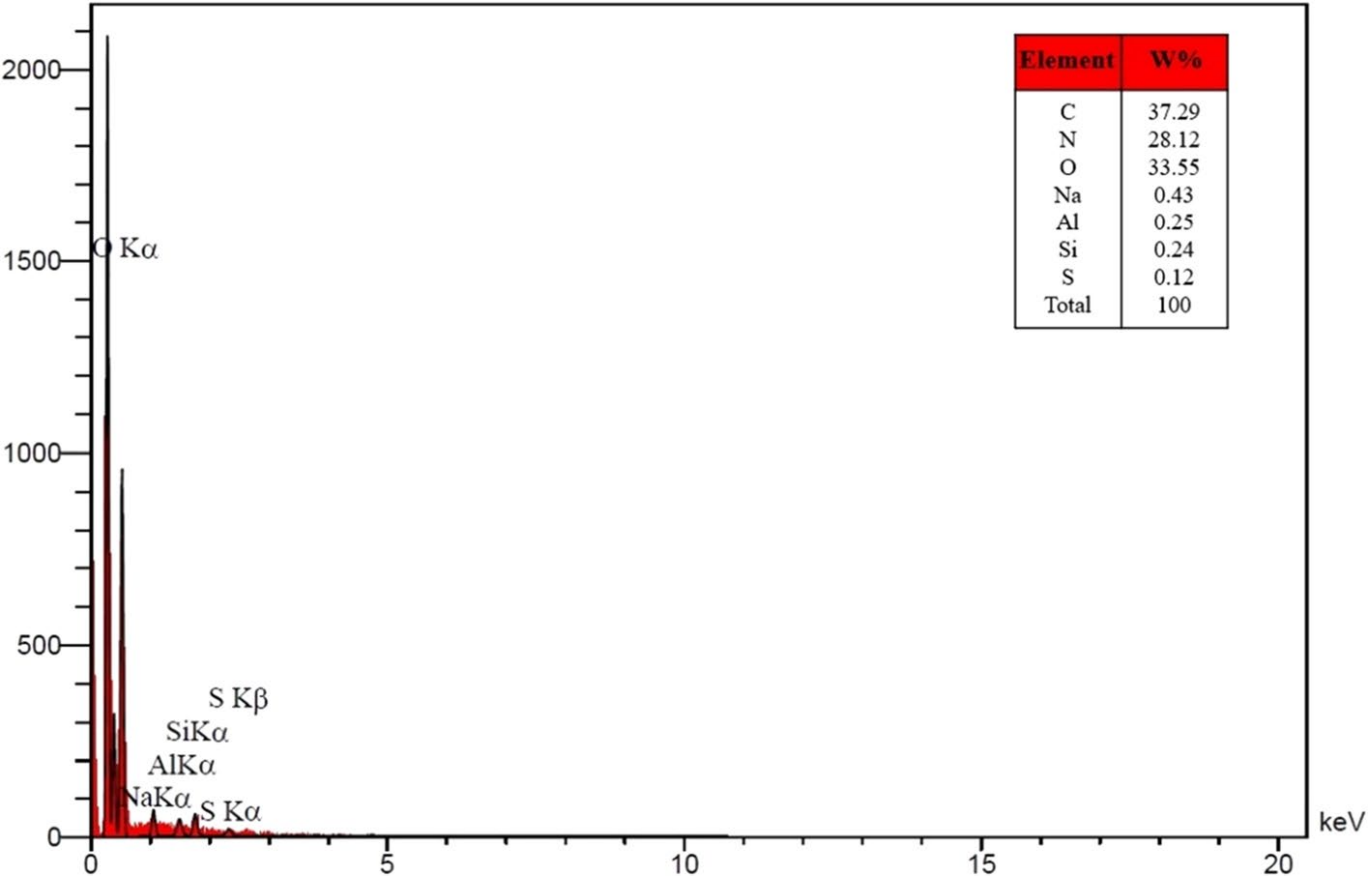
EDS patterns of the as-prepared hydrogel GQD/MMT_7_.

Figure 7 displays the EDS spectrum of the hydrogel GQD/MMT_7_ nanocomposite hydrogel. The EDS analysis provides information regarding the chemical composition and purity of the prepared nanocomposite hydrogel. The composition of the hydrogel GQD/MMT_7_ is determined through the EDS analysis, revealing the following elemental percentages: carbon (C) 37.29%, oxygen (O) 33.55%, nitrogen (N) 28.12%, Sodium (Na) 0.43%, aluminum (Al) 0.25%, Silicon (Si) 0.24%, and sulfur (S) 0.12%. The presence of oxygen (O) and carbon (C) elements indicates the incorporation of GQD and salep in the hydrogel nanocomposite semi-IPN structure. Additionally, the presence of silicon (Si), aluminum (Al), and sodium (Na) elements confirms the presence of MMT within the hydrogel nanocomposite semi-IPN. The observed peaks in the spectrum validate the successful integration of GQD/MMT within the polymer matrix, supporting the effective incorporation of GQD/MMT into the hydrogel structure.

### Mapping analysis of the hydrogel GQD/MMT_7_

**Figure 8:**
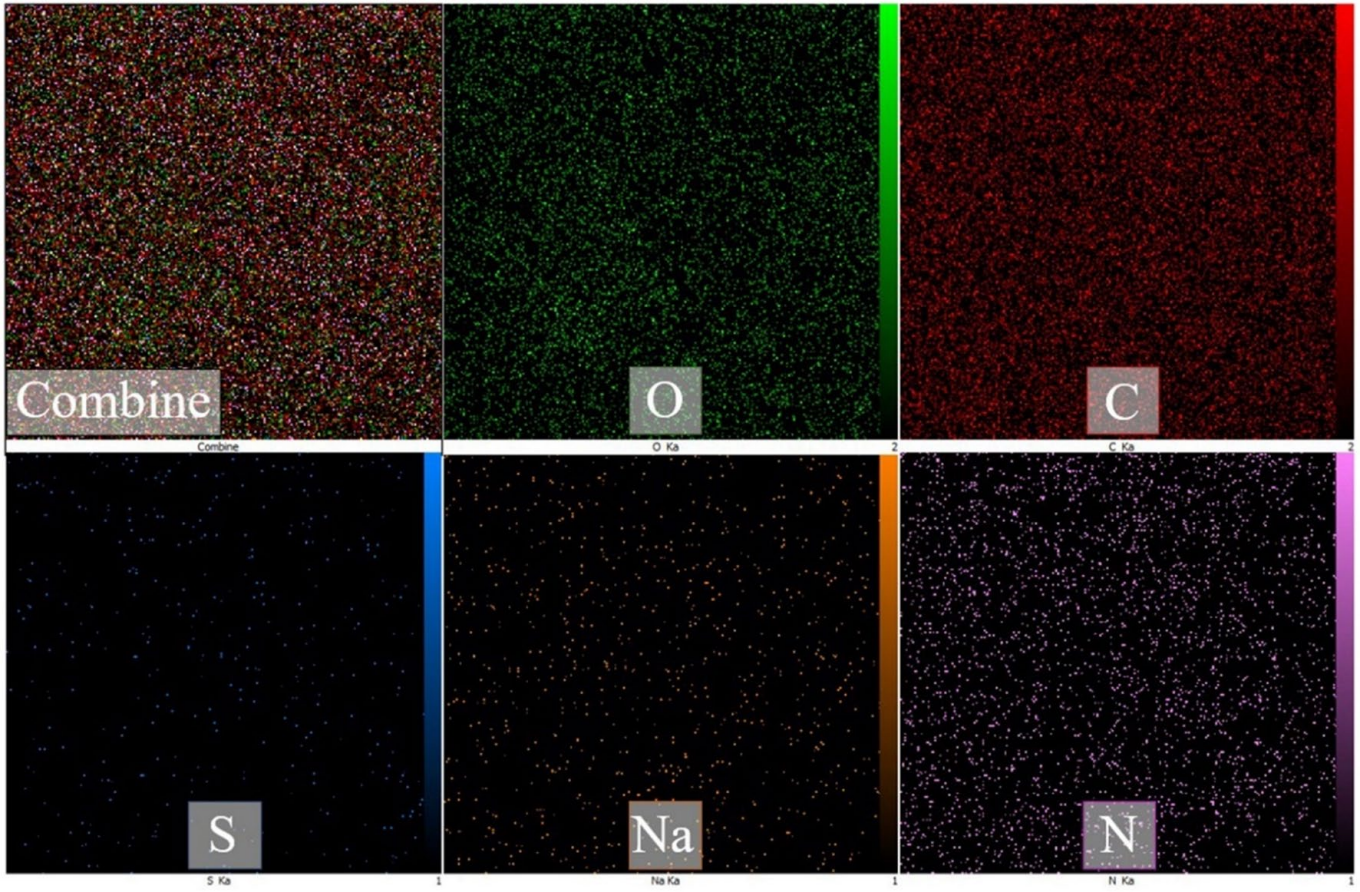
The elemental mapping images of hydrogel GQD/MMT_7_ (The green color represents regions enriched with oxygen (O), red represents carbon (C), orange represents sodium (Na), purple represents nitrogen (N), and blue represents sulfur (S)).

Figure 8 exhibits the mapping analysis results of the new and used nanoabsorbent. This analysis provides information about the density and distribution of constituent elements within the hydrogel GQD/MMT_7_ structure. The mapping analysis displays different colors representing specific elements within the nanocomposite hydrogel. The distribution of these elements appears to be well dispersed throughout the nanocomposite hydrogel, indicating a homogeneous distribution within the structure. This suggests that the elements O, C, Na, N, and S are well incorporated and distributed within the hydrogel GQD/MMT_7_ composite.

### Swelling behavior

**Figure 9:**
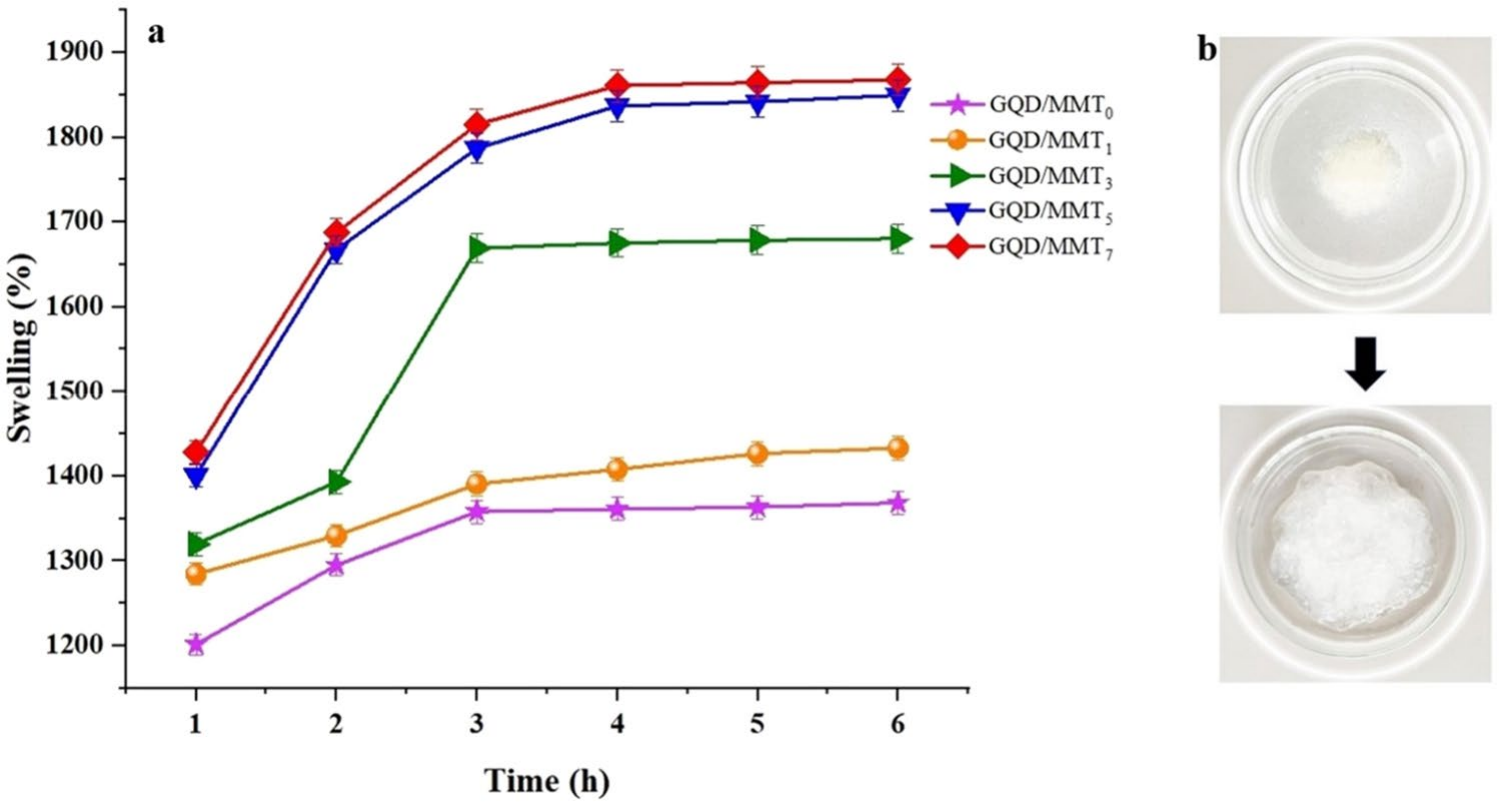
The swelling ratio of the hydrogels was evaluated at a specific time from 1 to 6 h (**a**) and freeze-dried hydrogel and swollen hydrogel in water (**b**).

Figure 9 shows the swelling rate of the hydrogel reinforced with GQD/MMT nano-absorbent for 6 hours. The results indicate that the swelling ratio of the preformed gel particles depends on their percentage composition. During the first 3 hours, the hydrogel and hydrogel nanocomposite exhibited an increased swelling rate, demonstrating the excellent performance of the hydrogel. As time progresses, the swelling capacity of the hydrogel reaches equilibrium. The desired swelling rate increases with increased GQD/MMT additive percentage. This indicates that the GQD/MMT nano-absorbent fundamentally affects the gel's structure and properties. The GQD/MMT nano-absorbent possesses a high specific surface area, ion exchange capability, and unique swelling ability. These factors improve the nanocomposite hydrogels' water absorption capacity and cumulative release rate. Overall, the incorporation of GQD/MMT nano-absorbent enhances the water absorption capacity and cumulative release rate of the nanocomposite hydrogels due to the unique properties of the GQD/MMT material.

### The effect of pH

**Figure 10:**
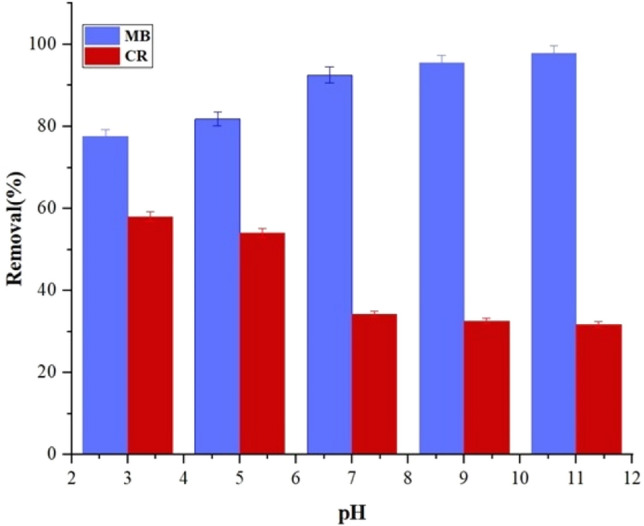
The pH effect on MB and CR adsorption.

The study investigated the effect of pH on the absorption of MB, as a cationic dye and CR, as an anionic dye, using 50 mg of the adsorbent and an initial concentration of 5 ppm. The results obtained from the effect of pH on MB and CR absorption with an initial concentration of 5 ppm and absorbent amounts of 50 mg for 7 wt% samples during 1 h at pH values of 3, 5, 7, 9, and 11 with a temperature of 25 °C were measured. Solutions of HCl and NaOH were utilized to adjust the pH. The obtained removal efficiency is shown in Fig. 10.

The highest removal percentage of the MB dye was observed at pH = 11. As the pH increased, the absorption of the MB dye by the adsorbent also increased, reaching its maximum at pH around 11. The increase in absorption at pH values greater than 7 can be ascribed to the absence of H^+^ ions. At pH values greater than 7, the concentration of H^+^ ions decreases significantly, the solution becomes more alkaline or basic, and the carboxylate groups (-CO_2_^-^) tend to be deprotonated. This enhancement in absorption is due to an increase in electrostatic attraction. Both chemical and physical interactions influence the adsorption process of dyes on the hydrogel. The surface charges of the adsorbent change with the pH, which significantly affects the removal of ionic dyes^64^.

In this case, the hydrogel surface contains amino groups (–NH_2_) and hydroxyl groups (–OH), which facilitate the establishment of hydrogen bonds between the hydrogel and dyes. Therefore, the absorption behavior is optimal under neutral pH conditions.

The removal rate of CR decreases as the pH increases. In alkaline pH, the carboxylate groups (–CO_2_^−^) lose their H^+^, and this causes a great repellence with anionic dyes due to electrostatic repulsion. The highest removal efficiency of CR dye is achieved in acidic pH conditions. In acidic pH, protonation of active sites and increased positive charge density on the adsorbent surface lead to a high removal efficiency in this case.

**Figure 11:**
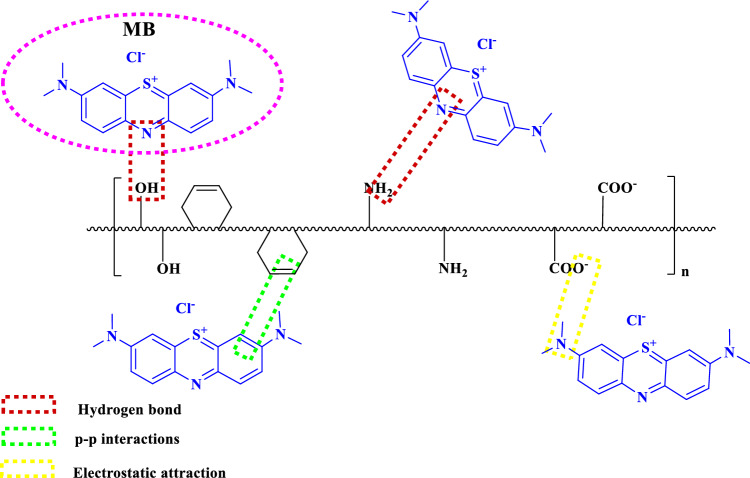
Adsorption mechanism between the hydrogel nanocomposite semi-IPN and MB.

The improved absorption characteristics can be attributed to the unique arrangement of GQD and MMT on the sheet, which increases the surface area and provides access to oxygen-containing groups. This increased surface area and availability of functional groups likely contribute to the enhanced removal efficiency. Furthermore, the positively charged dye molecules can undergo various interactions with the GQD/MMT nanocomposite, including hydrogen bonding, electrostatic interactions, and π-π interactions (Fig. 11). These interactions can further enhance the absorption capacity of the nanocomposite for dyes. To confirm these hypotheses, the removal rates of both anionic and cationic dyes were investigated, as shown in Fig. 8^45^.

### Contact time effect

**Figure 12:**
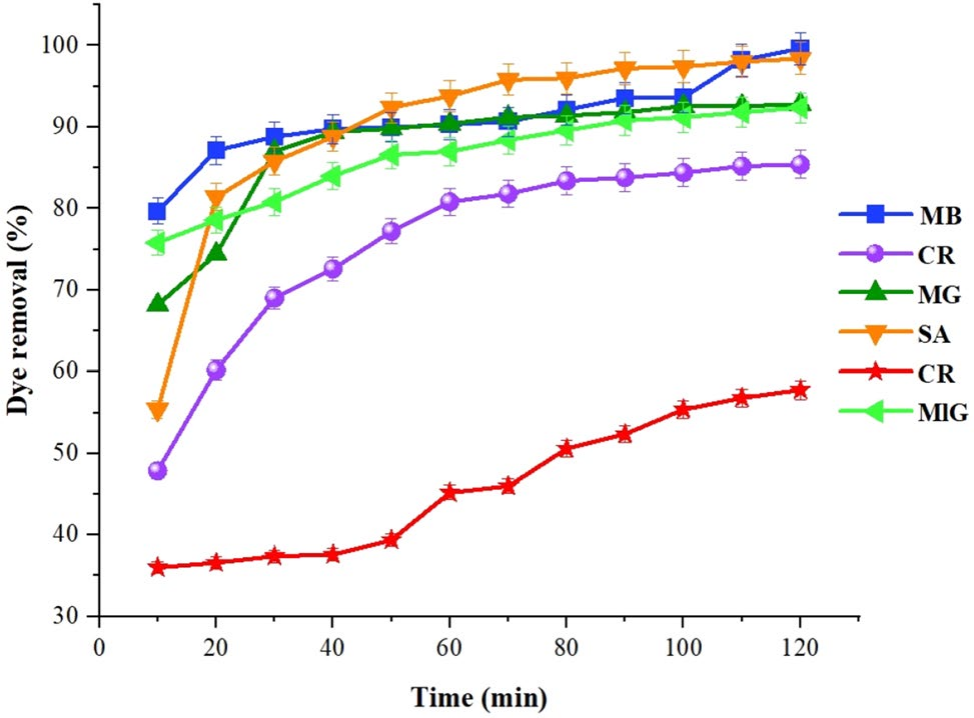
Contact time effect on dye removal efficiency of sample GQD/MMT_7_.

The concentration and dosage of the absorbent were also taken into consideration. The contact time was varied from 10 to 120 min, and the absorption efficiency was evaluated. The results indicate that the adsorbent's color absorption process occurs rapidly and shows a steep slope. For instance, in the case of the cationic dye MB, the removal efficiency was 79.6% at 10 min, which increased to 90.30% at 60 min and finally reached 99.64% at 120 min. Figure 12 illustrates the effect of contact time on the absorbent and demonstrates the trend of increasing removal efficiency with longer contact times.

#### Additive dosage effect

**Figure 13:**
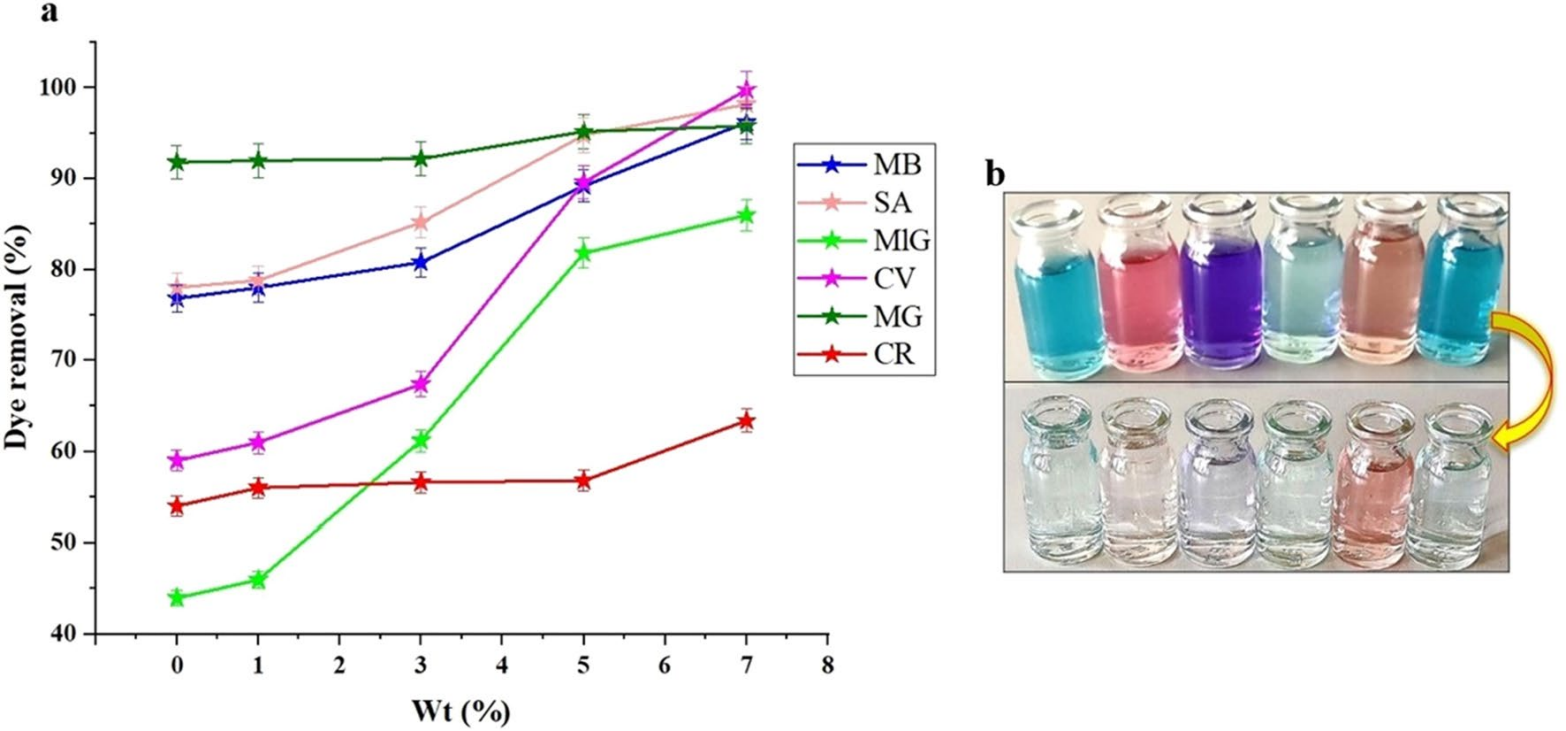
Effect of adsorbent dosage (**a**) and images before and after adsorption at room temperature (**b**).

The adsorbent dosage determines the capacity of the adsorbent for a given initial concentration of the colored solution. The aim is to achieve efficient utilization of the adsorbent while minimizing costs. In this study, 25 mL of colored solution with an initial concentration of 5 ppm was mixed with varying amounts of adsorbent (0–7 wt%), and the absorption of each sample was assessed over 90 min. The results obtained from the absorption process, using a consistent pH and contact time, demonstrate that the absorption efficiency increases with higher doses of the adsorbent within the optimal time frame of the process (Fig. 13a). The incorporation of GQD/MMT nano adsorbent into the polymer matrix leads to an increase in the specific surface area and internal pores of the adsorbent. This, in turn, enhances the absorption efficiency and removal of dyes. For instance, when the hydrogel GQD/MMT_3_ was added, the removal efficiencies (R%) for MB, SA, MlG, CV, MG, and CR were 80.8, 85.2, 61.2, 67.4, 92.2, and 56.6%, respectively. These values reflect the relatively low number of active sites available for absorption compared to the significant presence of pollutants. However, the addition of the hydrogel GQD/MMT_7_ resulted in significantly increased removal values, with R% values for MB, SA, MlG, CV, MG, and CR reaching 96.2, 98.2, 86, 99.8, 95.8, and 63.4%, respectively. This suggests that a 7 wt% concentration of GQD/MMT nano adsorbent is the most suitable percentage by weight. Furthermore, Fig. 13b compares the color before and after absorption under optimal absorption conditions. It is observed that the color becomes brighter after the absorption process.

### Adsorption isotherm models

Adsorption isotherms are crucial in evaluating the performance of adsorbents^65^. To gain insights into the equilibrium between adsorbent molecules on the surface and molecules in the bulk solution, isotherm data can be analyzed using three commonly used models: Langmuir, Temkin, and Freundlich isotherm models^66^.

#### Langmuir isotherm

The Langmuir isotherm suggests that adsorption on the solid surface occurs as a monolayer with homogeneous sites. It also implies no further adsorption occurs once all the active sites are covered with dye molecules. Equations (3) and (4) represent the mathematical expressions for the Langmuir isotherm, which describe a saturated single-layer model^67^.

3 $${{\text{q}}}_{{\text{e}}}=\frac{{{\text{q}}}_{{\text{m}}}.{{\text{k}}}_{{\text{L}}}.{\text{C}}}{(1+{{\text{k}}}_{{\text{L}}}.{{\text{C}}}_{{\text{e}}})}$$

4 $$\frac{{\text{Ce}}}{{{\text{q}}}_{{\text{e}}}}=\frac{1}{{{\text{k}}}_{{\text{l}}}{.{\text{q}}}_{{\text{max}}}}+\frac{{{\text{C}}}_{{\text{e}}}}{{{\text{q}}}_{{\text{max}}}}$$

where *q*_*e*_ (mg.g^−1^) represents the amount of adsorbate adsorbed per unit mass of adsorbent at equilibrium, and *C*_*e*_ (mg.L^−1^) is the equilibrium concentration of the adsorbate in the solution after the adsorption process.

**Figure 14:**
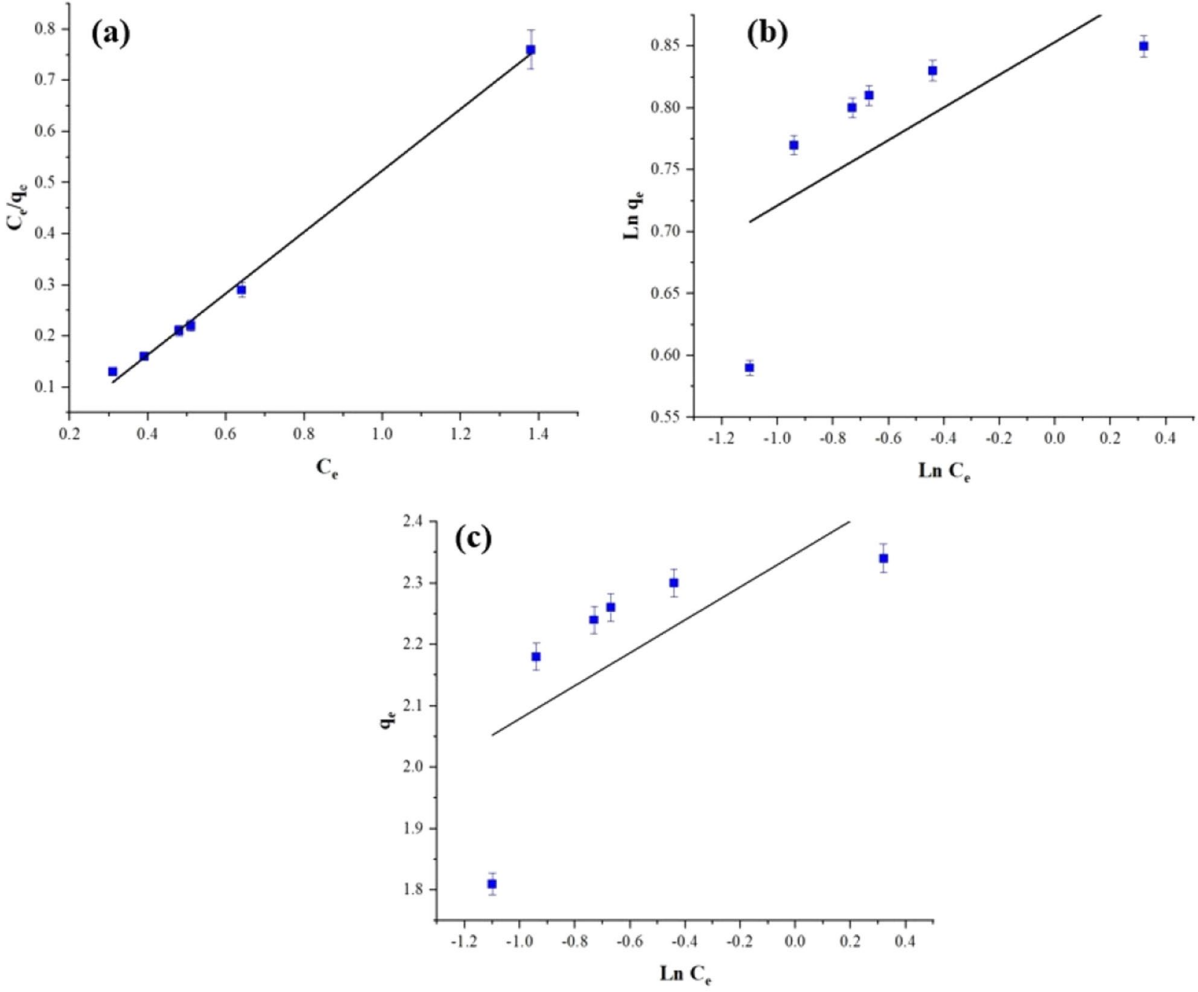
Langmuir (**a**), Temkin (**b**), and Freundlich (**c**), isotherms plots for MB.

The q_max_ and K_L_ constants for the Langmuir isotherm, estimated from the C_e_/q_e_ versus C_e_ graph, are presented in Fig. 14. Table 2 provides the Langmuir constants specifically for the MB dye.

**Table 2:** The isotherm-constants of (MB) adsorption process on semi-IPN hydrogel nanocomposite.

Temperature (°K)	Temkin model			Freundlich model			Langmuir model		
	K_T_ (Lg^−1^)	B (J mol^−1^)	R^2^	K_f_	n_f_	R^2^	q_max_ (mg/g^−1^)	K_L_ (Lmg^−1^)	R^2^
298	7.9	2.4	0.4908	12.18	0.66	0.4856	15.02	0.6	0.9967

The Langmuir adsorption isotherm is a better fit for the experimental data, as indicated by a higher correlation coefficient. The Langmuir model assumes maximum adsorption occurs when a single layer of adsorbate molecules on the adsorbent surface becomes saturated. Consequently, the adsorbed molecules do not separate from the adsorbent surface during adsorption.

#### Temkin isotherm

This model also indicates that the absorption heat decreases linearly with increased absorption quantity. The Temkin isotherm model is expressed in Eq. (5)^68^:

5 $${{\text{q}}}_{{\text{e}}}={{\text{BlnK}}}_{{\text{t}}}+{{\text{BLnC}}}_{{\text{e}}}$$

where *B* = *RT∕b*, *b* (J/mol), is Tamkin constant, which shows the heat of absorption, *T* (°K) is the absolute temperature, *R* (8.314 mol^-1^ K^-1^) is the gas constant, and *K*_*t*_ is the equilibrium binding constant.

The compliance constants can be determined by analyzing the graph of qe versus LnC_e_. Figure 14 illustrates this graph. The specific values of these constants for the Langmuir isotherm can be found in Table 2.

#### Freundlich isotherm

The Freundlich isotherm describes adsorption on a heterogeneous surface where the distribution of binding energies is non-uniform. It accounts for both heterogeneous and multilayer adsorption. Equation (6) represents the mathematical expression for the Freundlich isotherm^69^:

6 $${{\text{Q}}}_{{\text{e}}}={{\text{k}}}_{{\text{F}}}{{\text{C}}}_{{\text{e}}}^{1/{\text{n}}}$$

where *Q*_*e*_ (mg/g) is the amount of dye absorbed in equilibrium, *C*_*e*_ (mg/L) is the concentration of dye in equilibrium, *k*_*F*_ ((mg∕g) (L∕mg) ^(1∕n)^) Freundlich constant and intensity constant absorption.

This Freundlich model can be linearly expressed as Eq. (7)^70^:

7 $$\mathrm{Ln }{{\text{q}}}_{{\text{e}}}={{\text{Lnk}}}_{{\text{F}}}+\left(\frac{1}{{\text{n}}}\right){{\text{LnC}}}_{{\text{e}}}$$

The *K*_*F*_ in the Freundlich isotherm is related to the adsorption capacity. It allows us to determine the intensity of the adsorbate-adsorbent interaction, represented by the surface inhomogeneity factor 1/n.

To obtain the Freundlich constants, *Ln(qe)* and *Ln(Ce)*, the graph can be analyzed as depicted in Fig. 14. The specific constant values for the Freundlich isotherm in the case of MB are provided in Table 2.

### Kinetic studies

Adsorption kinetics is essential in defining adsorption efficiency^71^, and pseudo-second-order and pseudo-first-order laws are commonly used^72^. The pseudo-first-order kinetic model (Eq. (8)) is as follows:

8 $${\text{Ln}}\left({{\text{q}}}_{{\text{e}}}-{{\text{q}}}_{{\text{t}}}\right)={{\text{Lnq}}}_{{\text{e}}}-{{\text{k}}}_{1}{\text{t}}$$

The linear form of the pseudo-second-order kinetic model (Eq. (9)) is expressed as follows:

9 $$\frac{{\text{t}}}{{{\text{q}}}_{{\text{t}}}}=\frac{1}{\left({{\text{k}}}_{2}{{\text{q}}}_{{\text{e}}}^{2}\right)}+\frac{{\text{t}}}{{{\text{q}}}_{{\text{e}}}}$$

**Figure 15:**
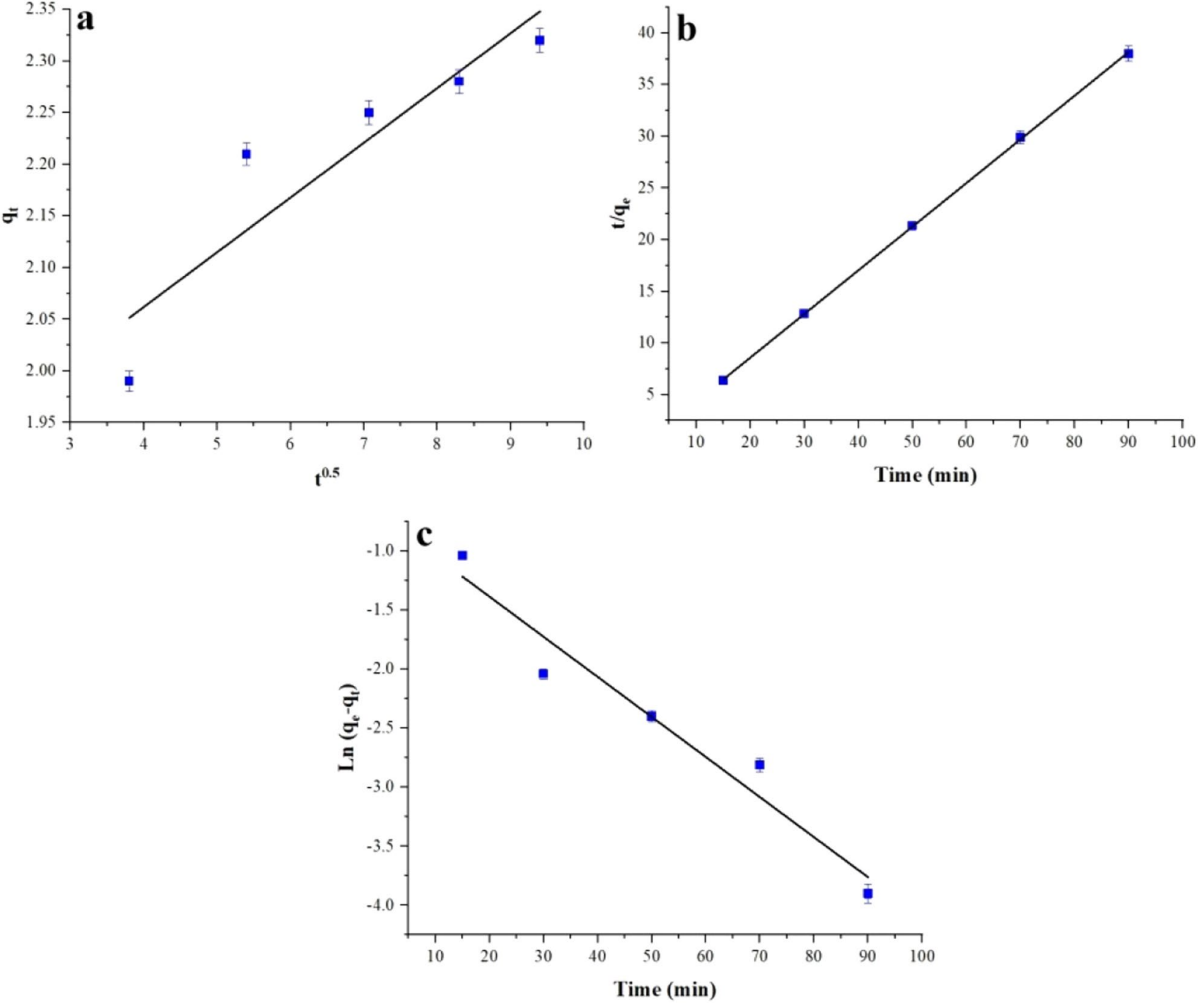
The intra-particle-diffusion plots (**a**), pseudo-second-order (**b**), and pseudo-first-order (**c**) for MB.

Moreover, intra-particle penetration is expressed by the Webbrs pore diffusion model (Eq. (10)), which is widely used to study the absorption mechanism.

10 $${{\text{q}}}_{{\text{t}}}={{\text{k}}}_{{\text{i}}}{{\text{t}}}^{1/2}+{\text{C}}$$

where *q*_*e*_ and *q*_*t*_ (mg.g^-1^) show the color absorbed on the adsorbent at time *t* (min), pseudo-first-order kinetic constant. *k*_*2*_ (g(mg.min)) is the pseudo-second-order kinetic rate constant. *k*_*i*_ and *C* are the intraparticle diffusion rate constant and the constant for the thickness of the boundary layer. The diagram of these equations for MB adsorption is shown in Fig. 15.

The constants associated with the pseudo-equations used in the kinetic studies for the MB dye can be found in Table 3. The results indicate that the pseudo-second-order model exhibits R^2^ values close to 1, surpassing other equations discussed in the context of kinetics. Therefore, compared to other kinetic equations, the pseudo-second-order model appears to be the most appropriate for describing the adsorption process of a synthetic adsorbent for dye removal. This suggests that the adsorption mechanism involves chemical absorption rather than physical absorption.

**Table 3:** The kinetic constants for MB.

Time (min)	Pseudo first order			Pseudo second order			Intra diffusion	
	k_1_	q_e_	R^2^	k_2_	q_e_	R^2^	k_i_	R^2^
15–90	0.039	2.01	0.949	0.34	2.34	0.9999	0.15	0.8396

### Thermodynamic studies

Temperature is an important and influential factor in color absorption, as it affects the adsorption process. Thermodynamic parameters can be used to interpret the changes in the amount of dye absorption with temperature. These parameters provide information about the adsorption process's feasibility, mechanism, and spontaneity. Examples of thermodynamic parameters include the enthalpy change (ΔH°), entropy change (ΔS°), and Gibbs free energy (ΔG°). By analyzing these parameters, one can gain insights into the thermodynamic aspects of the adsorption process and understand the energy changes and molecular interactions involved^73^. Modifications of the absorption length can be estimated using the following relations (11) and (12)^74^.

11 $${{\text{L}}}_{{\text{n}}}{{\text{k}}}_{{\text{d}}}=\frac{\Delta {{\text{s}}}^{0}}{{\text{R}}}-\frac{\Delta {{\text{H}}}^{0}}{{\text{R}}}\cdot \frac{1}{{\text{T}}}$$

12 $$\Delta {{\text{G}}}^{0}=\Delta {{\text{H}}}^{0}-\mathrm{T\Delta }{{\text{S}}}^{0}.$$

The equilibrium distribution coefficient is *k*_*d*_, the temperature is ${\text{T}}$(°K), and *R* is the universal gas constant equal to 8.314 J mol^−1^ K^−1^.

**Figure 16:**
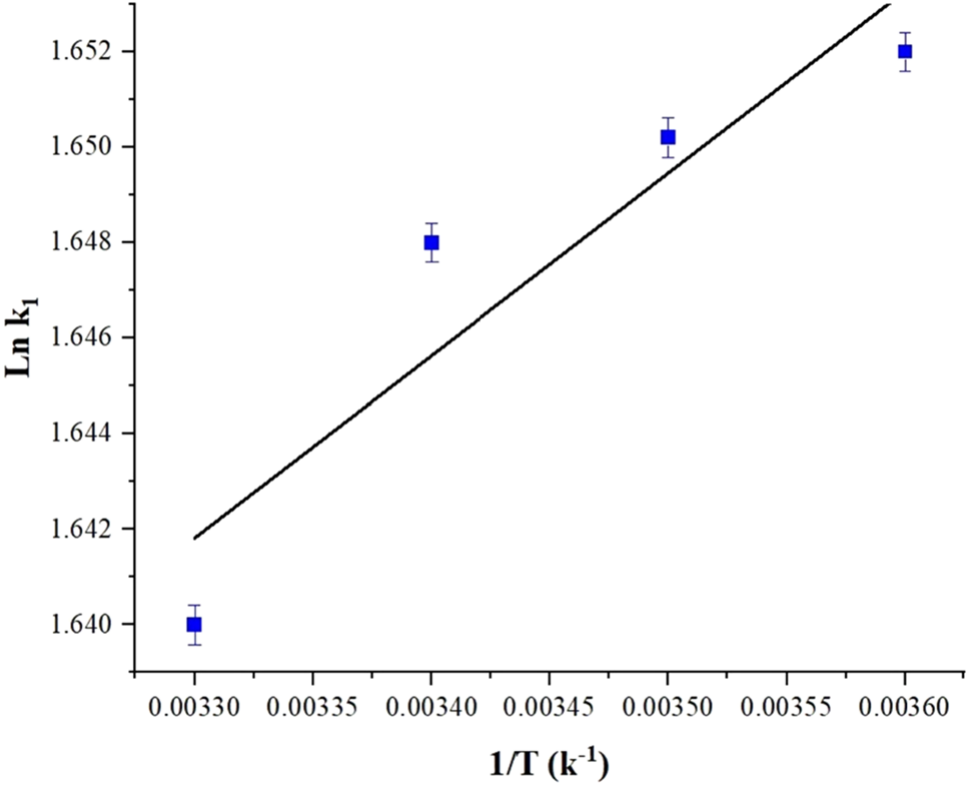
The thermodynamic plot of MB adsorption.

Figure 16 illustrates the thermodynamic diagram of the MB dye adsorption. The adsorption of MB onto the adsorbent is an endothermic process, as evidenced by the positive value of ΔH°, which is determined to be 1.054 kJ mol^−1^. The observation of increased adsorption capacity with increasing temperature further supports this finding. This suggests an affinity of the MB dye for the active sites of the adsorbent. The results demonstrate that as the temperature increases from 278 to 303 °K, the values of ΔG° become increasingly negative, ranging from − 4.6549 to − 5.0735 kJ mol^−1^. This confirms the spontaneity of the MB dye absorption onto the synthesized adsorbent, as indicated in Table 4.

**Table 4:** Thermodynamic parameters of MB adsorption.

Dye	T (°K)	ΔG (kJ mol^−1^)	ΔH (kJ mol^−1^)	ΔS (kJ mol^−1^ K^−1^)
MB	278	− 4.6549	1.054	0.016
	288	− 4.8223		
	298	− 4.9898		
	303	− 5.0735		

## Study of reusability

**Figure 17:**
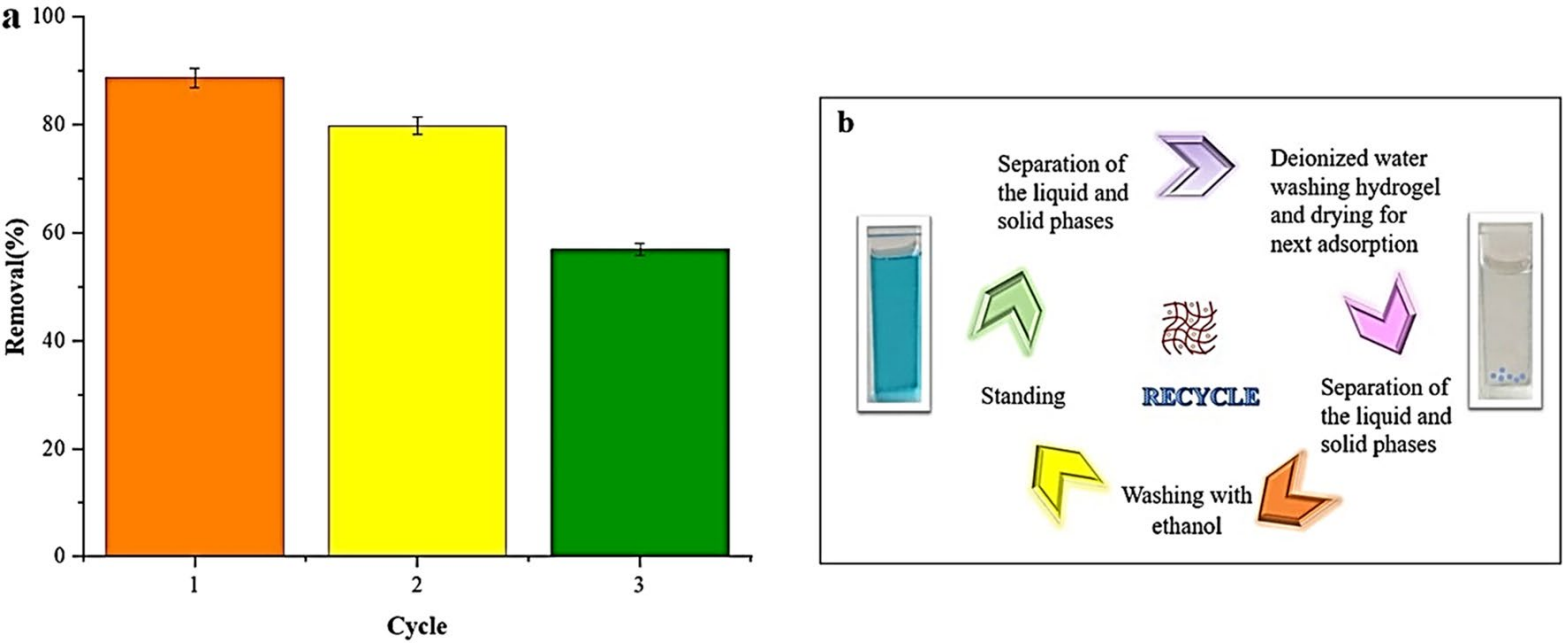
Reusability study of the hydrogel containing GQD/MMT_7_ at room temperature (**a**) and adsorption–desorption and reusability experiment procedure (**b**).

The ability to recycle and reuse the absorber was investigated as one of the most essential factors in the design of the absorber. The adsorbent was immersed in HNO_3_, HCl, and CH_3_CO_2_H, 24 h after adsorption. However, the maximum regeneration was obtained using ethanol. Hence, the results of the other two regeneration agents were not presented. To investigate the usability of the prepared adsorbent, ethanol was used as the solution to study the desorption effect. A cationic dye MB was used to examine the prepared hydrogel GQD/MMT_7_ reusability. Figure 17b shows the reconstruction test method. Figure 17a also explains that the adsorbent can be used several times without significantly reducing its efficiency, even after three cycles.

## Other absorbents

Table 5 presents a comparison between the current performance of the hydrogel nanocomposite semi-IPN adsorbents for removing organic dyestuffs from wastewater and other cases previously reported in the existing literature. The current adsorbent's absorption capacity is very high, surpassing the capacities reported in other studies. Additionally, the present adsorbent can remove different colors simultaneously and be utilized for several cycles, indicating its potential for efficient and sustainable dye removal from wastewater.

**Table 5:** Comparison between references and this work.

Adsorbents	Adsorbates	Removal (%)	Ref.
GQD	CV	99.1	^75^
CS/N-GQD	MB	84	^52^
Poly(AAm-co-AMPS)/Na-MMT	CV	80	^76^
Natural clay	CR	94	^77^
MMT-Fe_3_O_4_/PES	MB	92.8	^3^
Hydrogel nanocomposite GQD/MMT	MB	96.2	Current study
	SA	98.2	
	MG	95.8	
	CV	99.8	
	MlG	86	
	CR	63.4	

## Conclusion

In summary, the study evaluated an adsorbent synthesized from salep biopolymer-based hydrogel substrate with varying percentages of GQD/MMT nano adsorbent additive for the removal of MlG, MB, MG, CR, SA, and CV dyes from wastewater. The successful synthesis of the GQD/MMT nano adsorbent additive was confirmed through techniques such as FTIR. FESEM analysis showed that increasing the weight percentage of GQD/MMT led to an increase in the adsorbent's holes and surface area. TGA and EDS analyses confirmed the successful synthesis of the semi-IPN nanocomposite hydrogel structure. The adsorbents exhibited rapid swelling ability with increasing weight percentages of the GQD/MMT additive. The removal efficiency of the dyes was investigated, and after 90 min of adsorption, high removal percentages were achieved: SA (98.2%), MlG (86%), CV (99.8%), MB (96.2%), MG (95.8%), and CR (63.4%). The optimal amount of the GQD/MMT additive was 7 wt%. The adsorption of MB was fitted using the pseudo-second-order model and Langmuir isotherm. Thermodynamic calculations demonstrated endothermic and spontaneous adsorption processes from 278 to 303 K. The ability to regenerate and reuse the adsorbent was tested, and the best recovery of MB dye was achieved using ethanol. Overall, the semi-IPN nanocomposite hydrogels demonstrated effective adsorption of cationic dyes and showed potential for practical applications in treating dye-contaminated wastewater.

## Data Availability

All data have been given in the article.
